# How does one find a satisfying career? An open letter to people <20 years old (None of whom probably read the *Croatian Medical Journal*, so feel free to pass this on.)

**DOI:** 10.3325/cmj.2022.63.207

**Published:** 2022-04

**Authors:** Charles H. Calisher

We have three grandchildren who are at tender ages. One entered college a few months ago and seems to have expected that his life goals (the road to fame, fortune, and happiness) should have been clear by now. Another is half way through college and wonders the same thing. The third has graduated from college, has had many excellent experiences, and is wondering what is next for him. They are intelligent, stay out of trouble, and are pleasant, charming, and hard workers. Their parents have raised them properly and given them good advice. Their grandparents have also given them good advice. The problem is that they are not at all comforted by anything anyone close to them (us) has said. Instead, after long, thorough, quiet, and excellent explanations as to how the world works (or doesn’t work), after they have been given examples that might convince them to relax a little bit, enjoy school, and not try to peek over the horizon, they say, “But what should I do?” To respond, “I just told you”, does not help anyone.

As adult readers of this discourse will recognize, “the best laid plans of mice and men often go awry” (a line from a poem by the Scottish poet Robert Burns). Most of us know from our own experiences that exact planning for or even trying to predict one’s life is rarely successful and not any fun. That would be like someone giving you lists of what to expect as gifts for your future birthdays and Christmases.

I decided to ask various friends, colleagues, and other people (a well-known author, a world-class virologist, an attorney, a university chancellor, and others) how they ended up with the careers at which they had been so successful. I told them I wanted to gather their stories for this column and that they would remain anonymous, except to me; I have done that. With a single exception, the brief comments that follow were the result of this non-statistically significant poll. I sort of plagiarized the exception.

1. A Colorado native, she struggled with mathematics throughout her formative years due to a learning disability. She graduated from college without taking algebra. However, algebra was a prerequisite of another college where she intended to matriculate for a Master’s degree in natural resources. By her perseverance, and studying self-paced classes, she continued to learn about the love of her life, mathematics, especially statistics. Once enrolled, she thrived. Her integrated degree included classes in range science, recreation science, watershed management, and forestry. “Those four disciplines prepared me very well for two different professions,” she said. She now manages a ranch where she raises llamas and has taught middle and high school science and mathematics for more than 30 years.

2. “On a traveling summer camp experience in the southwest when I was 12, I had to select a project, as did other kids, and mine was describing and collecting spiders and scorpions. This cured me of any primordial fears I had of these creatures, and allowed a new-found appreciation of the natural world. This experience awoke me to science and biology, eventually leading to a career path. In high school I took a biology class. The teacher believed in hands-on projects. I spent a year figuring out inheritance of fruit fly characteristics. This started to focus my interest in biology from the naturalist experience described above into more sophisticated underlying biological mechanisms. Both were transformative experiences. It would not matter if the subject matter had been different, ie, could be almost any field from literature to astronomy. I tried to find an interest by being open to new experiences. If the interest is there it will blossom and grow and will lead to immense satisfaction. In college I took a graduate course in herpetology while a freshman, just to see what it was about. I guess I was drawn to scary creatures (spiders…snakes). The course was in a world-class museum. I loved the glass cabinets with stuffed animals I had never seen before, the smell of formaldehyde, the tangible feeling of history. I did a year-long study of the hearing apparatus of salamanders – it was eventually published, my first paper. Everyone I told thought this was a pretty foolish way to spend a year of work. My feeling was that the subject didn’t matter, it was the discipline of scientific observation and doing a thorough job of it that mattered. Moreover, it was a great deal of fun! I eventually went on to medical school.”

3. “At first, it was the summers. Needing money for college, I worked as a laborer, mingling with the “lifers,” shoveling mud, digging ditches, loading gravel, and far worse. By 16, I knew as a certainty that I wanted to use brains instead of brawn for my livelihood.

Then it was an inspiring professor. I was floundering in my studies of aeronautical engineering due to lack of interest and application, so I switched to business, merely to proceed toward a degree.

Fortunately, business law was required. Even more fortunately, my professor loved the law and ignited a love of the law in me and other students, so I made the decision to attend law school as soon as my military obligations were satisfied. I loved the law and was loathe to retire at age 79. I suggest you find something you love doing.

Three of my best friends followed me into the grueling summer labor market and were all equally inspired, becoming, respectively, a radiologist, owner of the heavy tool division of an international company, and Senior Vice-President of a successful investment banking firm.”

4. “I always knew, from about the age of 12, that I wanted to be some sort of writer or some sort of “nature guy”. Then I had some great teachers of English and only good biology teachers, so I pointed toward writing. I wrote and published a novel when I was 20 years old but I wasn’t meant to be a novelist because I had few deep experiences to draw on. Then I read Charles Darwin, and a number of other great writers in the realm of science, and I realized that I wanted to explain to ordinary readers the amazing work that those people had done. I transformed into a nonfiction science writer, and have had the most extraordinary opportunities for forty years, meeting and writing about the works of Jane Goodall, Edward O. Wilson, Richard Dawkins, Karl M. Johnson, Beatrice Hahn, Charlie Calisher, and many others.” [Author’s note: “Charlie Calisher”?]

5. “I grew up in Wilmington, Delaware. My father worked for the duPont company, and I graduated from Pierre S. duPont public high school. From my teenage years, I wanted to be veterinarian, so in my senior year in high school I applied for a pre-veterinary program at a nearby college in a nearby state. While in college, I had an opportunity to spend two summers as a chore boy on a working ranch in Wyoming. When I watched the wranglers handle the horses and cattle, I realized that I was a “city boy” and knew nothing about these large animals. Also in college, I worked with a local veterinarian, immunizing thousands of chickens on poultry farms. It was hot, smelly, and dirty work, so I decided to switch to pre-medicine instead.

In my senior year I applied to various medical schools and was accepted at one in Philadelphia. Also in my senior year, I decided to go West and applied for a one-year rotating internship at San Francisco General Hospital. That was a great experience.

After finishing my internship, on a lark [for fun] I applied for a pediatric residency at Gorgas Hospital in the old Panama Canal Zone, because I was interested in tropical diseases.

This was in 1962-63, during the height of the Vietnam War, and the military needed doctors. Interns and residents began receiving draft notices even before finishing. By this time, I had a wife and infant daughter and didn't want to go to Vietnam, so I enlisted as a Commissioned Officer in the US Public Health Service. I was assigned as a Peace Corps physician in Northeast Brazil. This experience changed my life. I got to travel widely in Brazil, learn Portuguese, and learn about new cultures. I also fell in love with the tropics.”

6. “I grew up on a small livestock/dairy farm in northern Illinois. I really just wanted to stay on that farm, but my father (who, having never finished high school, was determined that his children would go to college and have a better life) told me if I didn’t go to college, I had to move out. So, I went to college [Author’s note: He moved out to do this.] to study biology so I could go to veterinary school and then return to the farm. Well, I needed a way to help pay for veterinary school and enrolled in a joint veterinarian-Master’s degree program that came with a tuition waiver. At the time, this seemed like a foolish reason to enroll in a graduate program, but what I discovered was that I loved being in the lab.

After a while, I changed direction and from the farm and a career as a large animal veterinarian I moved to Purdue University, where I obtained a PhD and residencies in pathology and toxicology. The toxicology PhD was almost an accident – Purdue had an open position in a combined toxicology-pathology program, and that’s where I ended up. Without planning for it all, I found myself best qualified for a career in the pharmaceutical industry and I went to work for what was then the pharmaceutical division of a chemical company. I didn’t like the industry work very much and when an opportunity opened for an assistant professorship at Oregon State University, I jumped at it – mostly because it was in Oregon and my wife and I wanted to live in the west and hike and do other outdoor activities; again, not the most professional reason to choose a job. When Oregon’s voters elected to reduce taxes dramatically, all of us in the veterinary school received our letters of 1-year notice. I had my first NIH grant and Colorado State University had an opening, so I took that. Two years later, I had just been given tenure and was the chair of our department’s faculty advisory committee when our chair resigned abruptly. I agreed to serve as acting chair for four months while we did a search. Well, I discovered the budgets were in need of repair and I had no real idea how to fix that so I just opened the department’s books at a faculty meeting and asked for ideas. Apparently, that cry for help was interpreted as an act of transparency and my colleagues asked the Dean to appoint me into the chair role. The Dean and I got along well and he asked me to serve as the associate dean for research. That introduced me to central administration and a couple of years later when the vice-president for research retired, he asked me to apply for his job. I thought then (and still do), that my role was to have an internal candidate in the pool in case the search went badly, which apparently it did because at the end of the second round of interviews they asked me to interview and I was offered the job. Having not planned very well, I found myself offered something I didn’t really want but I didn’t perceive that I had a way to say No. Thus, I moved into university administration. Four years later our new president asked me to serve as provost, the chief executive officer of the university, and when he resigned four years later the Board appointed me to his role. In that time, I guess I’d say I enjoyed every job I ever had, was never looking for the next one, and was never sure I had the right combination of skills and experience to take the next step. Nonetheless, I thought there were some things I could help with, I knew how to hire good people to help, and I cared about the place I worked. I’ve come to believe that combination is pretty powerful because your decisions aren’t about you – it’s about creating an environment that allows so many other people to succeed and excel. I imagine that if you went back and said to the boy on the farm that you’ll end up as a university professor, I don’t think he’d have believed you. There certainly wasn’t a plan behind it. I simply did my best at each step, tried to keep an open mind about new opportunities and as I look back now, it has been a pretty satisfying ride and one that I’m proud of.”

So, there it is. Life is not predictable. Do not think you will make firm plans and stay with them. Simply do what you like at the time and if what seems like a better opportunity appears – take it. Eventually you will be doing what you like. The salary does not matter.

